# First-principle calculations of sulfur dioxide adsorption on the Ca-montmorillonite

**DOI:** 10.1038/s41598-022-24737-x

**Published:** 2022-11-24

**Authors:** Zhi-Jie Fang, Chang-Hui Song, Mei-Ling Liu, Bo Li, Shi-Kai Lin, Xiong-San Lin, Xiang Zhou, Qiu-Zhi He, Man Mo

**Affiliations:** 1 School of Electronics Engineering, Guangxi university of Science and Technology, Liuzhou, 545006 People’s Republic of China; 2 School of Civil Engineering and Architecture, Guangxi University of Science and Technology, Liuzhou, 545006 People’s Republic of China; 3 Tiandong Haorun New Material Technology Co., Ltd, Baise, People’s Republic of China; 4 College of Medical, Guangxi University of Science and Technology, Liuzhou, 545006 People’s Republic of China

**Keywords:** Environmental sciences, Materials science, Physics

## Abstract

According to the serious problem of sulfur dioxide pollution, montmorillonite is one of the effective ways in gas pollution control because of its excellent absorption properties. One of the fundamental questions is to fully understand sulfur dioxide absorption mechanism of montmorillonite. In this study, using the first-principle methods, we studied the adsorption characteristics of Ca-montmorillonite in the presence of $\hbox {SO}_{{2}}$. The adsorption energy and elasticity constants as a function of the adsorption capacity were also studied. The calculated results show that bridge site is the most stable adsorption site for $\hbox {SO}_{{2}}$ with the adsorption energy of − 140 meV. As adsorbent, Ca-montmorillonite is a clay with layer-structure, most of bond lengths(such as Al–O, Mg–O, Si–O, and H–O) does not obviously change. As adsorbed gas, the O–S–O bond angle of adsorbed $\hbox {SO}_{2}$ change from $119.50^\circ$ to $115.32^\circ$. The volume and adsorption energies of Ca-montmorillonite almost increase linearly with increasing $\hbox {SO}_{{2}}$ adsorption. By calculating the montmorillonite elasticity constants under different adsorption capacity, we found that the elasticity constant C33 which perpendicular to the crystal face, with the maximum changes from 450 to 326 GPa. In addition, Young’s modulus,bulk modulus and shear modulus significantly decrease with the increasing adsorption. The calculated results will not only help to understand the physical and chemical of montmorillonite but may also provide theoretical guidance for dealing with the problem of gas pollution.

## Introduction

Sulfur dioxide emissions have substantial impacts on atmosphere. Disasters caused by sulfur dioxide such as acid rain and haze seriously endanger people’s health and life. Therefore, sulfur dioxide is considered as the major pollution gas and one of the goals of controlling and achieving environmental protection^1^. The technology of $\hbox {SO}_{2}$ adsorption and storage can provide a medium-term solution to mitigate environmental impacts. Particularly, adsorption and storage have, by far, been the two most studied parts of the environmental technology chain. As a result, many investigators have studied experimentally the adsorption and storage of $\hbox {SO}_{2}$ using chemical and physical treatments^2^. However, most of the treatment technologies have many disadvantages such as complicated process or incur high costs. Mineral processing methods have the advantages of low cost, good results and no secondary pollution, which are the most important research directions for pollution treatment.

As a possible low-cost adsorbent, clay minerals, in particular, have received much attention in the storage of $\hbox {SO}_{2}$ taken from contaminated air. As one knows, montmorillonite is among the most abundant clay minerals. The experimental results show that clay minerals have strong adsorption capacity, which is due to the excellent adsorption performance^3^. Wang studied on the adsorption of clay minerals, using montmorillonite as a raw material to prepare a porous heterostructured mineral adsorbent with surface functionalization, and found that its adsorption capacity for toluene could reach up to 257.2 mg/g^4,5^. Plenty of researches on montmorillonite are to analyze the properties of montmorillonite to adsorb different kind of gas^6–9^. Through experimental research, it was found that the sequence of adsorption capacity of $\hbox {C}_{{2}}$ $\hbox {H}_{{2}}$ and $\hbox {CO}_{{2}}$ gas by different cation exchange montmorillonites, which indicates that the cation radius, interlayer distance and surface area of montmorillonite influences the gas adsorption. As we know, montmorillonite has the 2:1 interlayer structure, and the effect of absorbing gas molecules between layers is also remarkable. Therefore, the use of montmorillonite for the reduction of sulfur dioxide emissions become the new aspect of environmental protection^10^.

In contrast to the extensive experimental studies that have been carried out in the last decade, we found that understanding the interaction of the montmorillonite and the sulfur dioxide molecules was one main task for the researchers in environmental protection. To understand the complicated interaction of montmorillonite and sulfur dioxide, one need to know the detailed adsorption of montmorillonite. However, so far, there are very few theoretical reports on the sulfur dioxide absorption mechanism of montmorillonite^11–15^. In particular, we notice that an ab initio investigation of absorption mechanism in montmorillonite is still lacking. In addition, Ca-montmorillonite is a kind of montmorillonite which abundant in Guangxi, China. In order to make full use of the Guangxi province montmorillonite resources, in this paper, we report a series of first-principles for simulate and calculate the $\hbox {SO}_{2}$ adsorption of Ca-montmorillonite which most abundant montmorillonite in China. Furthermore, we analyze the changes in the structure of Ca-montmorillonite, adsorption energy, and elastic constant from a microscopic perspecitive. The aim of this paper also analyzes the changes in electronic structure and adsorption property of Ca-montmorillonite after $\hbox {SO}_{2}$ adsorption. We expect that the adsorption of sulfur dioxide first-principles results may be used to guide treatment of sulfur dioxide emissions by montmorillonite from a microscopic viewpoint. The remaining part of this paper is organized as follows. In “Methods”, we present our calculation methods. We give our results and discussion in “Results and discussion”. Finally, we briefly present our conclusion in “Conclusions”.

## Methods

**Figure 1:**
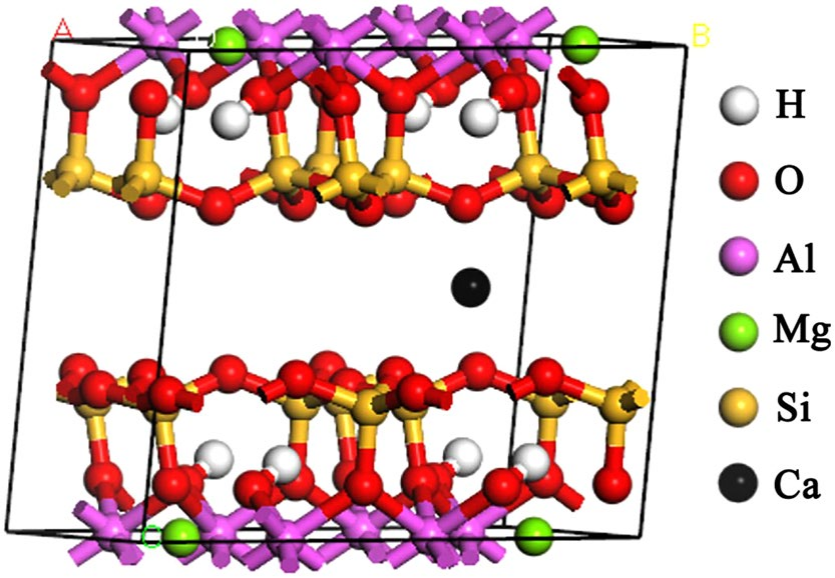
The structure of the Ca-montmorillonite.

Montmorillonite is a kind of clay mineral made up of a layer of octahedral aluminium oxide between two layers of tetrahedral sillicium oxides. We construct the montmorillonite calculation model with original formula $\hbox {Al}_{{2}}$ $\hbox {Si}_{{4}}$ $\hbox {O}_{{12}}$ $\hbox {H}_{{2}}$. In this study, in order to simulate and calculate the adsorption properties of montmorillonite, we studied the mechanism of sulfur dioxide adsorption on montmorillonite based on the calculations for the structure of the montmorillonite supercell. The suppercell composed of four united cells ($2 \times 2 \times 1$) was used to calculate the electronic structure and this includes 81 atoms. The calculation model of montmorillonite layer is shown in Fig. 1. Our calculations for the structure of montmorillonite are based on density-functional theory (DFT) within the local-density approximation (LDA)^16^ as implemented in the Vienna ab-initio simulation package (VASP)^17^ code through the use of the projector augmented wave (PAW) pseudopotentials^18^. All atomic positions are relaxed according to the calculated Helmann-Feynman force. Energy cut-off for the wave plan was set to 500 eV, and all atoms can be freely relaxed. The optimization of atomic geometries was performed via a conjugate-gradient algorithm until the residual force acting on atoms was less than 0.01 eV. The Monkhorst- Pack *k*-point which was set $2 \times 2\times 2$ (Monkhorst et al.^19,20^ was used to sample the Brillouin zone. 3*s* and 3*p* of Al, 3*s* and 3*p* of Si, 1*s* of H, 2*s* and 2*p* electrons of O are considered to be valence electrons in our calculations. In order to study the adsorption of sulfur dioxide molecules on montmorillonite, we will discuss two different aspects of adsorption: surface and interlayer adsorption. The adsorption energies of sulfur dioxide molecules on the montmorillonite are as follows^21^:

1 $$\begin{aligned} E_{ad}=E({montmorillonite}^{0}+nA)-E({montmorillonite}^{0})-nE(A) \end{aligned}$$

where $E({montmorillonite}^{0}+nA)$ is the total energy of montmorillonite supercell after adsorption, $E({montmorillonite}^{0})$ is the total energy of the montmorillonite without adsorption, *E*(*A*) is the energy of sulfur dioxide referenced to elemental gas, *n* is the number of sulfur dioxide molecules adsorption of the montmorillonite supercell structure, and $E_{ad}$ is the montmorillonite adsorption energy.

## Results and discussion

### Adsorption property

**Figure 2:**
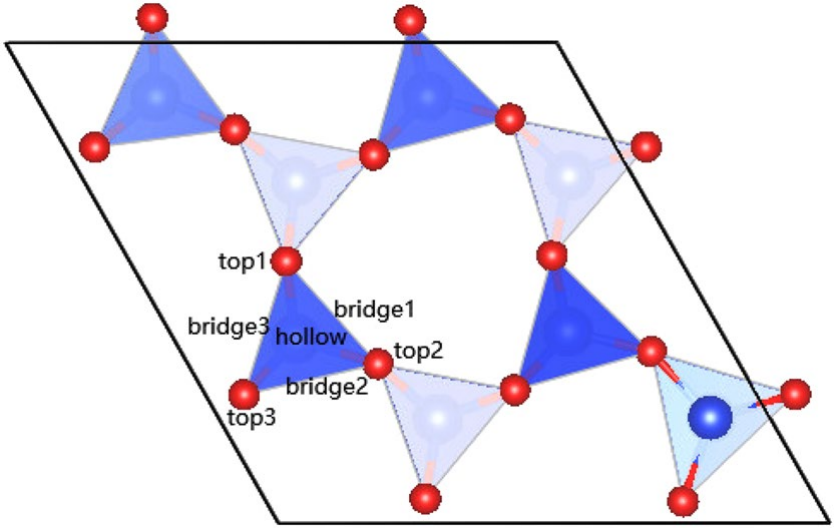
The adsorption site on the (001) surface of montmorillonite.

In order to study the adsorption of sulfur dioxide on a montmorillonite (001) surface, a vacuum layer of 15 Å has to be formed above (001) surface of montmorillonite. Three kinds of high-symmetry adsorption sites for sulfur dioxide were considered,including top site, bridge site,and hollow site, as shown in Fig. 2. During the surface calculations, we put the sulfur dioxide molecule above the topmost at the three chosen sites above, all the atoms in the calculated bulk positions are allowed to relax. When the sulfur dioxide is adsorbed on a clean surface of montmorillonite, the adsorption energies of the different adsorption sites are basically the same, which indicates that the difference of the adsorption surface is the factor determining whether the adsorption of sulfur dioxide is stable or otherwise. Overall, the adsorption energy of sulfur dioxide on a clean surface of montmorillonite is negative, indicating that the type of montmorillonite adsorption system belongs to heat stable adsorption. After relaxation, the calculated results show that, 2.82 Å was the most stable distance where adsorption energy was the minimum value at adsorption site. In addition, sulfur dioxide molecule prefers locating at 2.82 Å above the montmorillonite with the adsorption energy of − 0.13, 0.07, and − 0.14 eV calculated by Eq. (1) for top, hollow, and bridge sites, respectively. Thus, based on the result of adsorption energy, bridge and top site are the stable adsorption site for $\hbox {SO}_{{2}}$ on the surface of Ca-montmorillonite.

**Figure 3:**
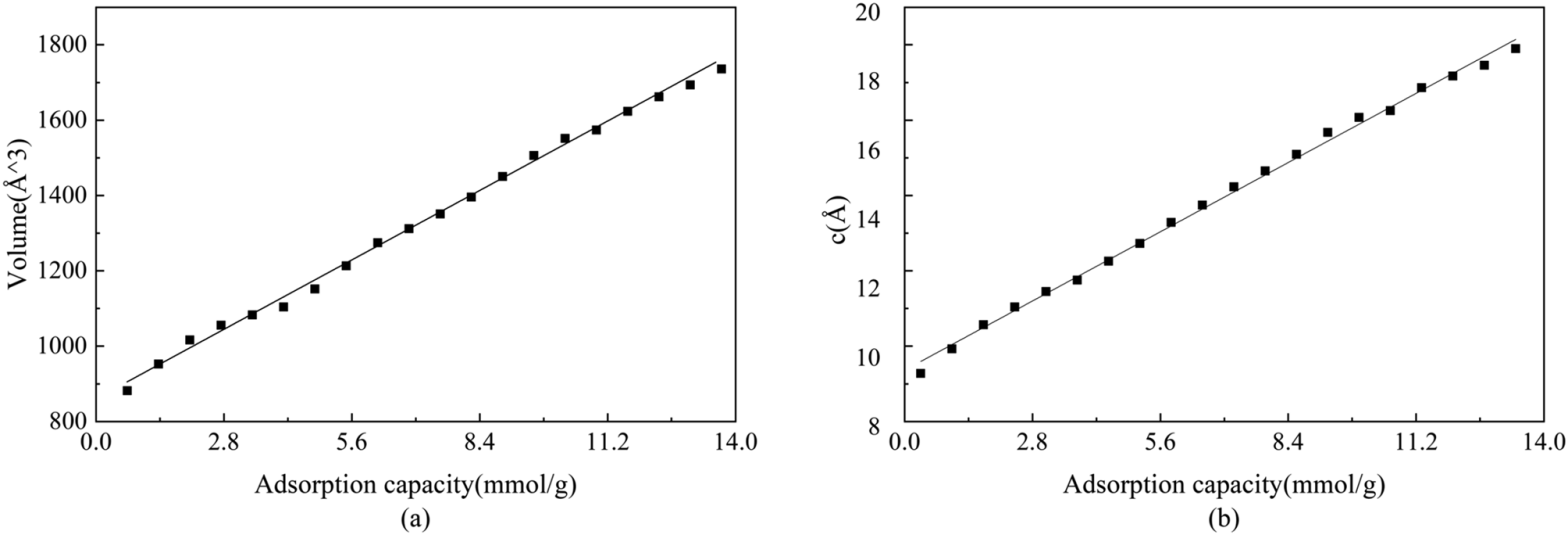
(**a**,**b**) Respectively represent the unit cell volume and (001) of montmorillonite after adsorption.

**Table 1:** The adsorption energies of different adsorption capacity within montmorillonite layer.

Adsorption capacity (mmol g$^{-1}$)	Adsorption energy (eV)	Adsorption capacity (mmol g$^{-1}$)	Adsorption energy (eV)
0.68	1.27	7.53	− 5.00
1.37	1.50	8.22	− 5.44
2.05	0.67	8.90	− 5.75
2.74	− 0.93	9.59	− 6.35
3.42	− 2.06	10.27	− 6.85
4.11	− 2.45	10.96	− 7.39
4.79	− 2.85	11.64	− 7.90
5.48	− 3.11	12.33	− 8.61
6.16	− 3.60	13.01	− 8.99
6.85	− 4.40	13.70	− 9.37

In view of particular structure of Ca-montmorillonite, layer adsorption become main adsorption of $\hbox {SO}_{2}$ within montmorillonite’s layer structure from the perspective of sulfur dioxide adsorption. In order to understand the adsorption properties of montmorillonite more accurately, we obtained the curve of volume change by structuring the montmorillonite (Fig. 3). It was found that the corresponding volume almost increase linearly with increasing $\hbox {SO}_{{2}}$ adsorption. In addition, it also can be clearly seen that the Ca-montmorillonite expanded along the (001) direction with the increase of adsorption capacity (Fig. 3b). This change is mainly because the volume of montmorillonite is caused by the increase in space between layers. It is consistent with the experimental result by X-ray diffraction (XRD). Volzone^22^ analyzed the structural and textural changes of montmorillonite after stepwise $\hbox {SO}_{{2}}$ adsorption. The X-ray diffraction diagram show that broadening of the (001) reflection indicated interlamellar disorder after repetitive $\hbox {SO}_{{2}}$ adsorption steps, while the structure of the layers was almost unchanged.

**Figure 4:**
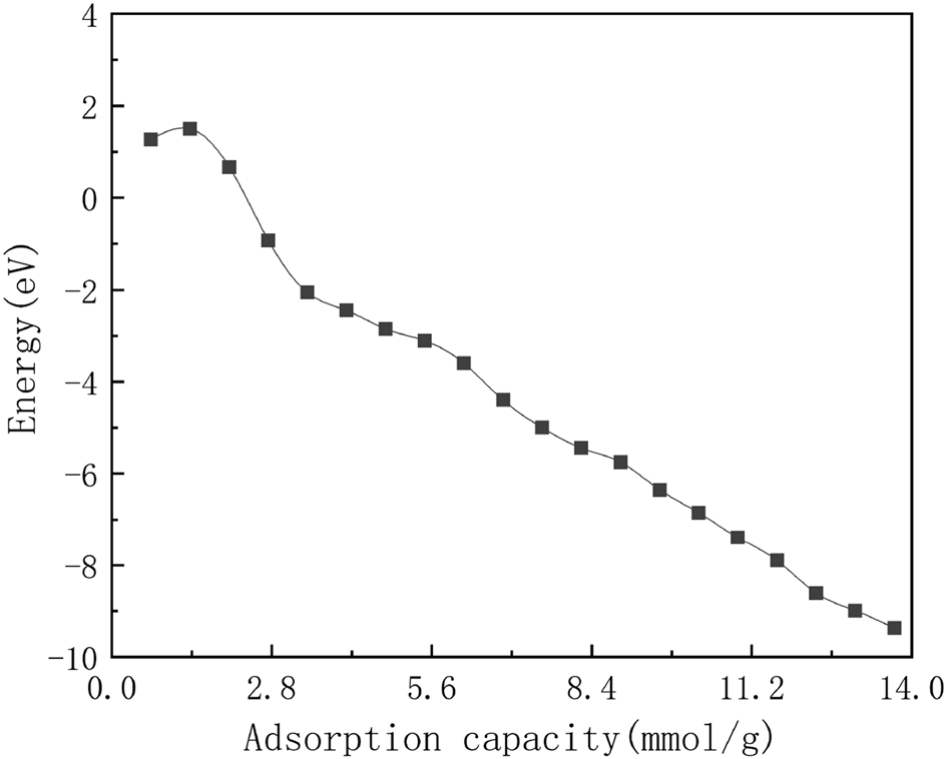
The adsorption energies of sulfur dioxide change according to adsorption capacity.

Table 1 lists the change of adsorption energies by different adsorption capacity. Furthermore, Fig. 4 shows the adsorption energies of sulfur dioxide change according to adsorption capacity. According to adsorption energy definition, a negative value of $E_{ad}$ indicates that the adsorption is exothermic (stable), while a positive value indicate endothermic (unstable) reaction. As shown in Fig. 4, with the early adsorbed period the value of $E_{ad}$ is positive and the later adsorbed period becomes negative. This change is mainly because when the adsorption behavior begins, the small quantities sulfur dioxide molecule may cause the structure of layers to become unstable. In contrast to the above volume results, our calculated result also show that the lower adsorption energies, and the better the adsorption property of Ca-montmorillonite.

### Sulfur dioxide and montmorillonite

**Figure 5:**
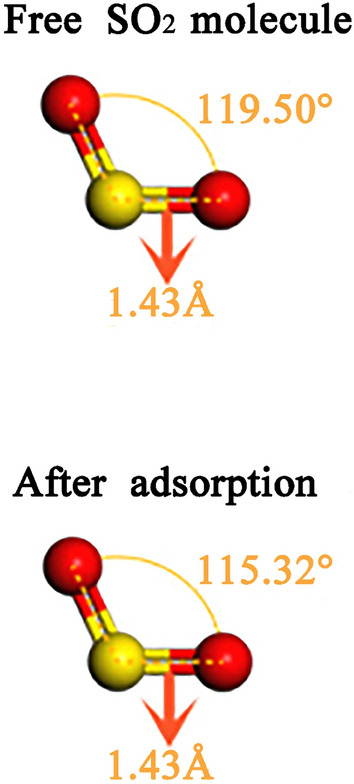
The structure of $\hbox {SO}_{{2}}$ free and after adsorption.

**Figure 6:**
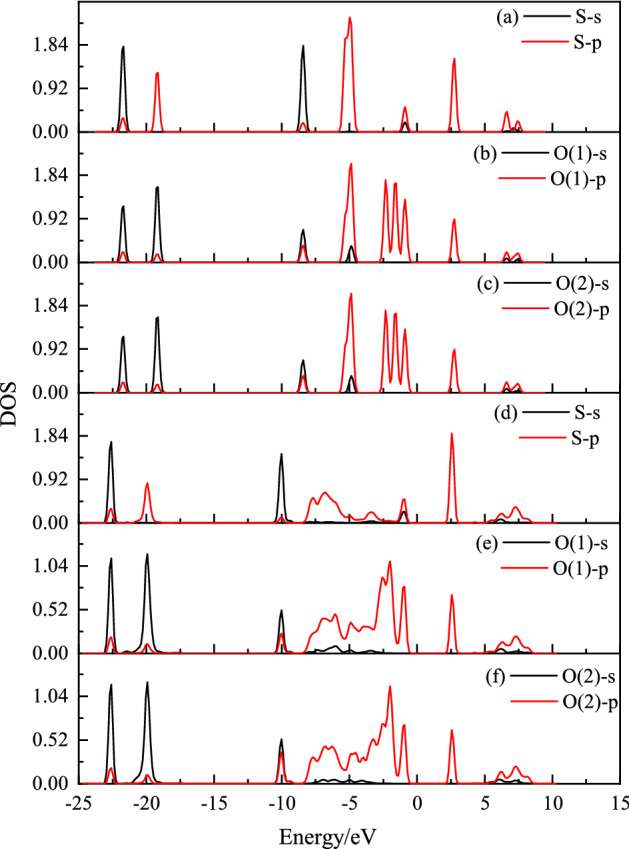
The change about orbital-resolved partial DOS for S and O atoms in $\hbox {SO}_{{2}}$ molecular after adsorption. (**a**–**c**) respectively represent orbital-resolved partial DOS for S, O(1), and O(2) atoms in $\hbox {SO}_{{2}}$ before adsorption; (**d**–**f**) respectively represent orbital-resolved partial DOS for S, O(1), and O(2) atoms in $\hbox {SO}_{{2}}$ after adsorption.

**Figure 7:**
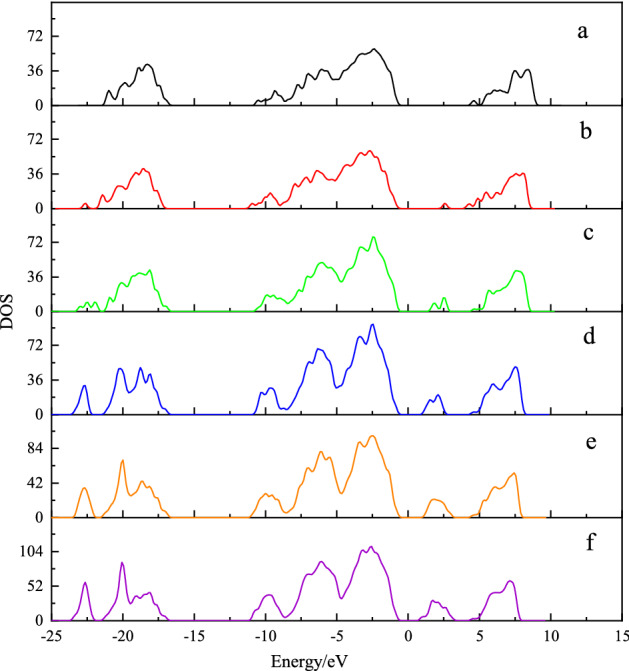
The change of total DOS in Ca-montmorillonite according to adsorption capacity. (**a**–**f**) Represent total DOS for Ca-montmorillonite according to the value of adsorption capacity are 0 mmol g$^{-1}$, 0.68 mmol g$^{-1}$, 3.42 mmol g$^{-1}$, 6.85 mmol g$^{-1}$, 10.27 mmol g$^{-1}$, and 13.70 mmol g$^{-1}$, respectively.

According to sulfur dioxide molecule, from the Fig. 5, it can be seen that the S–O bond length has no changed after adsorption, while the O–S–O bond angle of adsorbed $\hbox {SO}_{{2}}$ slight change from 119.5$^\circ$ to 115.32$^\circ$. With the help of density of states (DOS), one also can be better understood the sulfur dioxide and Ca-montmorillonite(seen in Figs. 6, 7). As can be seen from Fig. 6, the density of states peak mainly contribution to the 3*p* states of the S atom and the 2*p* states of the O atom before adsorption. Compared with the DOS of $\hbox {SO}_{{2}}$ before adsorption, the overall peak of partial density of states decreases and *p* states of S and O atoms spread from − 9.1 to − 0.2 eV, which means the DOS peak range spreads to lower energy region after adsorption. As shown from Fig. 7, one interesting result is that, the DOS peak appear at the 2.5 eV has increase after $\hbox {SO}_{{2}}$ adsorption. In contrast to Fig. 6, $\hbox {SO}_{{2}}$ adsorption is the main reason which *p* states of S and O atoms can be observed at 2.5 eV in bandgap region. While, the overall DOS profiles for Ca-montmorillonite are quite similar to that of perfect Ca-montmorillonite. In addition, we also discovered that the most of bond lengths and bond angle do not change substantially in Ca-montmorillonite after adsorption.

**Figure 8:**
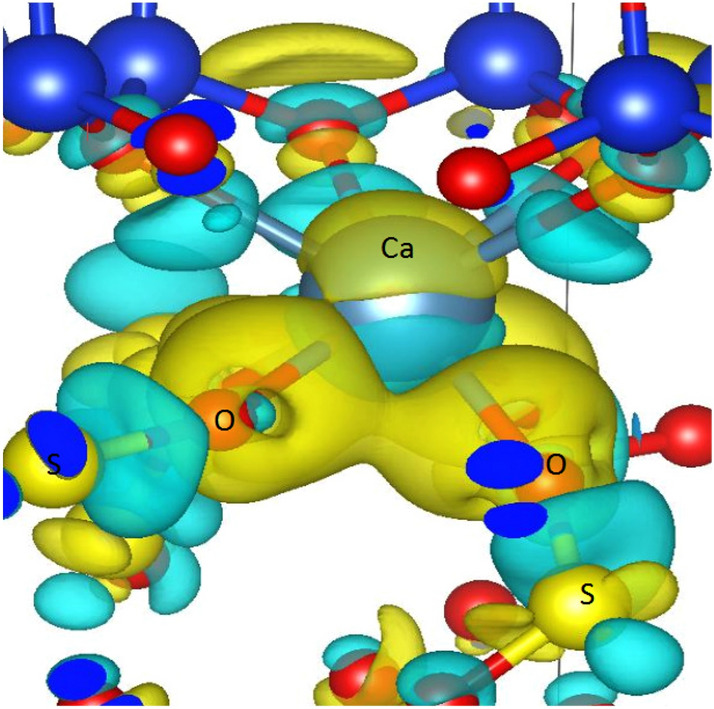
Differential charge density around Ca atoms after adsorption. Yellow color indicates electron accumulation and cyan color indicates electron depletion.

Furthermore, we plot the differential charge density around Ca atoms after sulfur dioxide adsorption. As shown in Fig. 8, some sulfur dioxide molecule around Ca atoms leads to the redistribution of valence electrons. As a result, an obvious electron accumulation can be observed, and the negative charge is formed around the Ca atoms. With this understanding, we emphasize that the formation mechanism of the electron accumulation is the DOS change of polar molecule $\hbox {SO}_{2}$.

### Elasticity constants

**Figure 9:**
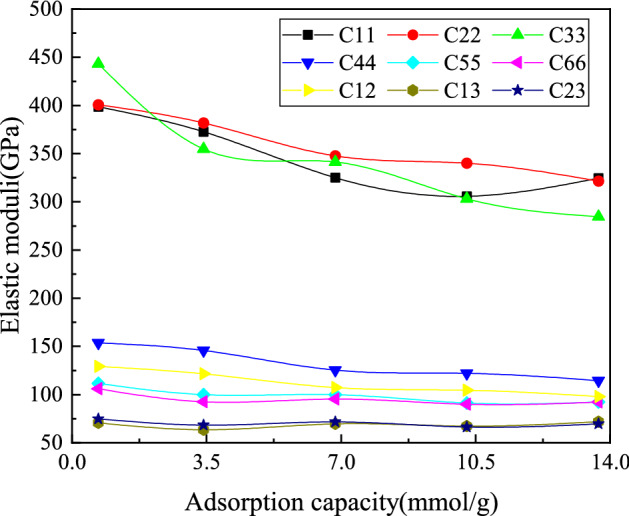
Elastic constants of montmorillonite change according to adsorption capacity.

In order to further investigation about the elastic mechanical behavior of montmorillonite after sulfur dioxide adsorption, the effects on the elasticity constant of montmorillonite according to adsorption capacity were further analyzed. Figure 9 show the elastic constants of Ca-montmorillonite under different adsorption capacity. It can be seen that the montmorillonite elasticity constants C11, C22 and C33 are significantly larger than other elastic constant, while C33 is larger than the elastic constants (C11, C22) parallel to the crystal plane. C11, C22 and C33 decreases with the increasing adsorption of sulfur dioxide molecules, especially the elasticity constant C33 which perpendicular to the crystal face (along z-axis direction), with the maximum changes from 450 to 326 GPa. C44, C55 and C66 correspond to the elastic constant of shear deformation, wherein the elastic constant C66 of the vertical plane in shear deformation is also smaller than the elastic constant C44 which parallel to the plane. The changing processes of montmorillonite elasticity constants C44, C55 and C66 for the shearing deformation decline slightly during the entire adsorbed process. In addition, the deformation elastic constants (C12, C13 and C23) are almost unchanged.

**Table 2:** The bulk modulus, shear modulus, Young’s modulus, B/G and Poisson’s ratio changed by adsorption capacity.

Adsorption capacity (mmol g$^{-1}$)	Bulk modulus *B*	Shear modulus *G*	Young’s modulus *E*	*B*/*G*	Poisson’s ratio
0.68	199.10	135.72	331.77	1.47	0.22
3.42	179.06	121.34	296.95	1.48	0.22
6.85	167.54	113.83	278.42	1.47	0.22
10.27	157.91	106.48	260.82	1.48	0.22
13.70	155.49	105.04	257.20	1.48	0.22

In order to understand the elasticity properties of montmorillonite more accurately, Table 2 lists the data of bulk modulus, shear modulus, Young’s modulus, B/G and Poisson’s ratio changed by adsorption capacity. The bulk modulus is greater than the shear modulus, indicating that montmorillonite has strong resistance to volume deformation and weak resistance to deformation. Young’s modulus can be used to characterize the stiffness of materials. It can be seen from the Table 2 that, with the increase of adsorption capacity, the smaller the young’s modulus, the weaker resist elastic deformation in montmorillonite. The changing trends of bulk modulus and shear modulus are similar with higher adsorption level. One interesting result shows that, Poisson’s ratio and *B*/*G* almost no change during the adsorption, which means Ca-montmorillonite keep plastic and brittle properties.

## Conclusions

In summary, we have studied the adsorption characteristics of sulfur dioxide in montmorillonite. The calculated results show that the O–S–O bond angle of adsorbed $\hbox {SO}_{{2}}$ change from 119.50$^\circ$ to 115.32$^\circ$ after adsorption, moreover, sulfur dioxide prefers locating at 2.82 Å above bridge site on the surface of Ca-montmorillonite. Ca-montmorillonite become expanded by along the (001) direction with the increase of $\hbox {SO}_{{2}}$ adsorption capacity. It also found that the corresponding volume, adsorption energies almost increase linearly with increasing $\hbox {SO}_{{2}}$ adsorption. By calculating the Ca-montmorillonite elasticity constants under different adsorption capacity, the calculated results show that, C11, C22, and C33 are greatly affected with the increasing adsorption of sulfur dioxide molecules. In addition, young’s modulus,bulk modulus and shear modulus significantly decrease with higher level of adsorption capacity. This results will not only provide theoretical support for the enhancement of development and application of Ca-montmorillonite in Guangxi, but will also provide a new path to resolve the problem of sulfur dioxide pollution in Guangxi, which has both fundamental academic and practical significance.

## Data Availability

The datasets used and/or analysed during the current study available from the corresponding author on reasonable request.
